# Supplementary material for: Continuous peripersonal tracking accuracy is limited by the speed and phase of locomotion
Source: Sci Rep. 2023 Sep 8;13:14864. doi: 10.1038/s41598-023-40655-y (PMC10491677; doi:10.1038/s41598-023-40655-y)
Supplement: Supplementary file 1 — Supplementary Figures. [file 41598_2023_40655_MOESM1_ESM.docx]

# Supplementary Figures

###
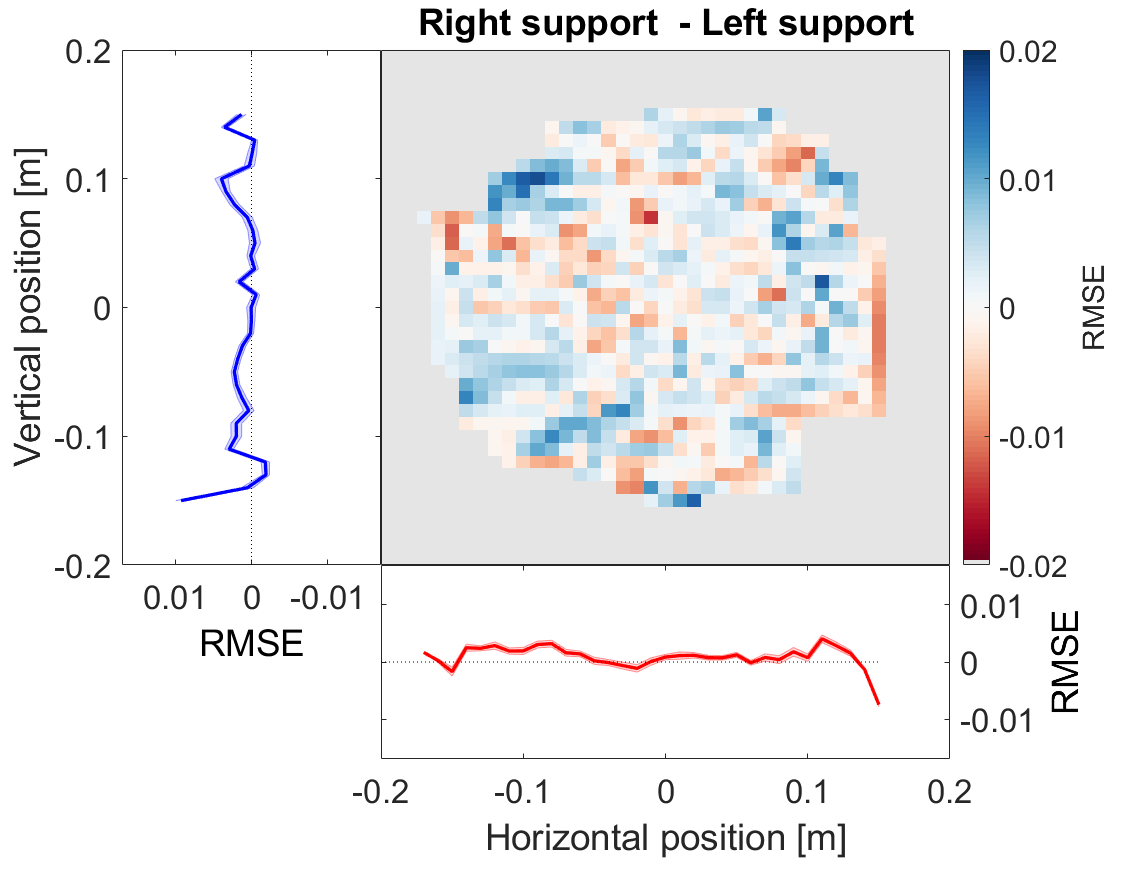
Supplementary Figure 1. No difference in peripersonal reach error when balanced on the left or right foot. All panels plot the difference in RMSE when comparing right support to left support stance. The heat map displays RMSE for each location. Left) Average difference in error at each position on the vertical axis (when averaging within all rows). Blue lines and shading display the average ± 1 SEM corrected for within-participant comparisons. Bottom) Average difference in error at each position on the horizontal axis (when averaging within all columns). Red lines and shading as in D. The magnitude of error when comparing left/right foot support, is never significantly different from zero after correcting for multiple comparisons.

###

###
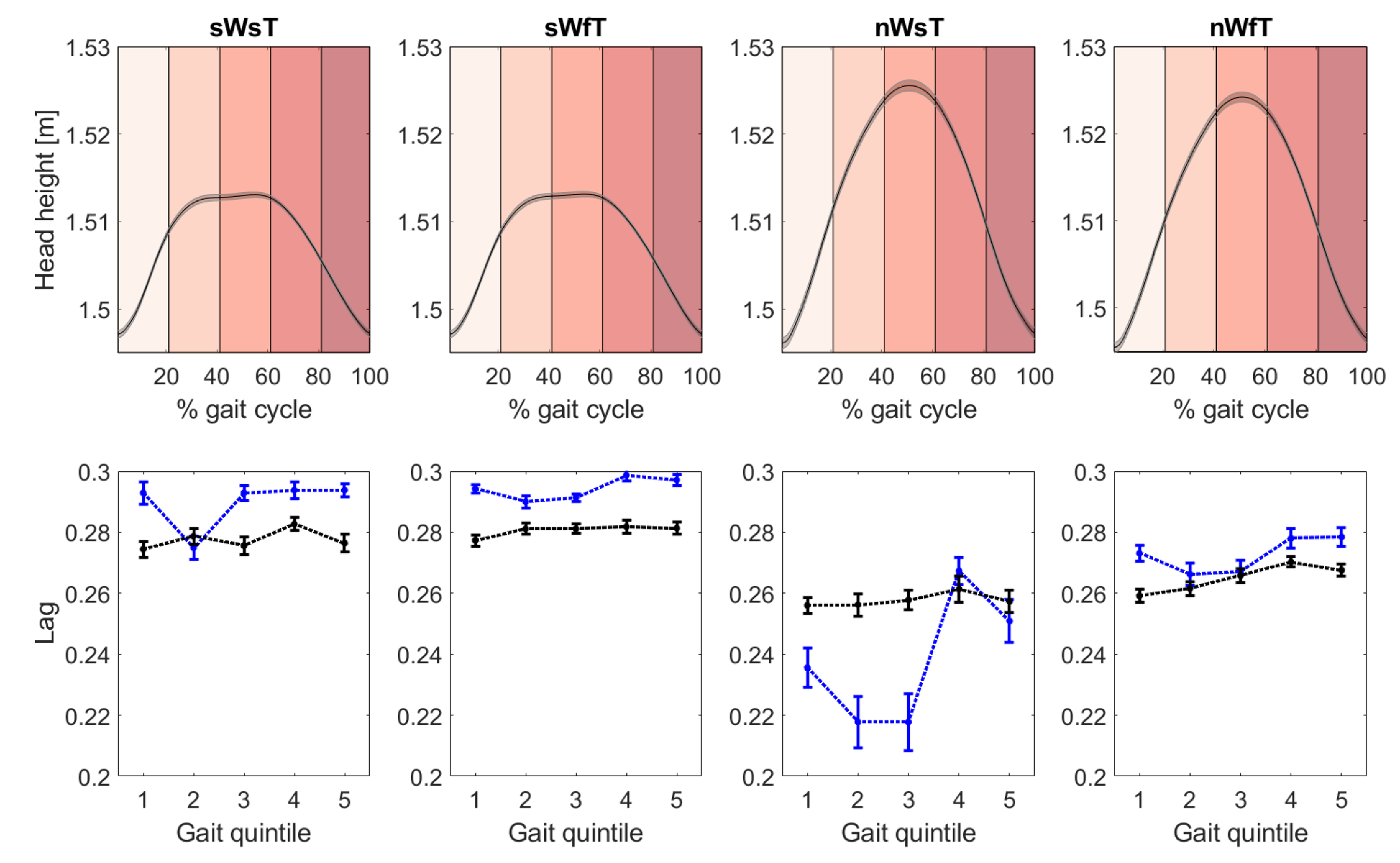


### Supplementary Figure 2. Windowed cross-correlogram per trial type. Blue and Black lines correspond to vertical and horizontal position shifts respectively. Error bars for 1 SEM corrected for within participant comparisons.
